# Optic neuropathy secondary to granulomatosis with polyangiitis in a patient with Graves’ disease: a case report

**DOI:** 10.1186/s13256-021-03207-4

**Published:** 2021-12-29

**Authors:** Miki Sato-Akushichi, Reiko Kinouchi, Naoko Kawai, Kenichiro Nomura

**Affiliations:** 1 Department of Ophthalmology, Asahikawa Medical University, Asahikawa, Hokkaido Japan; 2 Medicine and Engineering Combined Research Institute, Asahikawa Medical University, Midorigaoka Higashi 2-1-1-1, Asahikawa, Hokkaido 078-8510 Japan; 3 Department of Otolaryngology Head and Neck Surgery, Asahikawa Medical University, Asahikawa, Hokkaido Japan

**Keywords:** Hypertrophic pachymeningitis, Antineutrophil cytoplasmic antibody-associated vasculitis, Graves’ disease, Myeloperoxidase-antineutrophil cytoplasmic antibody, Drug-induced ANCA-associated vasculitis

## Abstract

**Background:**

Dysthyroid optic neuropathy is the most commonly suspected diagnosis of optic neuropathy in Graves’ patients; however, other causes need to be ruled out. We present a unique case of optic neuropathy secondary to hypertrophic pachymeningitis with antineutrophil cytoplasmic antibody-associated vasculitis, which was suspected to be antithyroid drug related.

**Case presentation:**

A 79-year-old Japanese male presented with acute visual loss in the left eye. He had a 24-year history of Graves’ disease and was taking methimazole. Best-corrected visual acuity was 0.8 in the right eye and light perception in the left eye, and relative afferent pupillary defect in the left eye was seen. Ocular movement was normal, and there were no findings explaining visual loss in intermediate optic media and fundus in the left eye. Contrast-enhanced magnetic resonance imaging demonstrated thickened dura mater. Tests for myeloperoxidase-antineutrophil cytoplasmic antibody, proteinuria, and hematuria were positive; pulmonary nodule lesions and a blood clot in the left lower leg were also found. After excluding the presence of diseases that could lead to hypertrophic pachymeningitis, we diagnosed optic neuropathy due to hypertrophic pachymeningitis with granulomatosis with polyangiitis—a subtype of antineutrophil cytoplasmic antibody-associated vasculitis. Since he had history of using methimazole, antineutrophil cytoplasmic antibody-associated vasculitis was considered as drug related. We started high-dosage steroid pulse therapy followed by 1 mg/kg body weight daily of oral prednisolone, and subsequently tapered. Methimazole was stopped. Best-corrected visual acuity recovered to 0.9, 2 weeks after starting treatment. Though myeloperoxidase-antineutrophil cytoplasmic antibody remained negative, the symptom relapsed 6 months after treatment initiation. We gave a second high-dose steroid pulse therapy followed by prednisolone tapered together with methotrexate. Remission remained, and using 4 mg/week methotrexate without prednisolone, myeloperoxidase-antineutrophil cytoplasmic antibody was kept within the normal limit until now, 4 years after onset.

**Conclusion:**

We present a case of optic neuropathy with hypertrophic pachymeningitis related to antineutrophil cytoplasmic antibody-associated vasculitis, which was suspected to be drug related. The patient had good visual recovery after quitting the drug and receiving immunosuppressive therapy with systemic steroids. Hypertrophic pachymeningitis with antineutrophil cytoplasmic antibody-associated vasculitis related to antithyroid drugs should be considered as a differential diagnosis for optic neuropathy in Graves’ patients in whom optic nerve compression is not obvious.

**Supplementary Information:**

The online version contains supplementary material available at https://doi.org/10.1186/s13256-021-03207-4.

## Background

In patients with Graves’ disease and concurrent optic neuropathy, dysthyroid optic neuropathy caused by either optic nerve compression or stretching is often at the top of the list of differential diagnoses [1, 2]. However, other causes of optic neuropathy, such as immunosuppressive drugs, radiation, infections, and inflammation, need to be ruled out [3]. Antineutrophil cytoplasmic antibody-associated vasculitis (ANCA-associated vasculitis) is characterized by inflammation of small to medium blood vessels, [4], and granulomatosis with polyangiitis (GPA), a subtype of ANCA-associated vasculitis, is one of the causes of inflammatory optic neuropathy [5]. Moreover, antithyroid drugs [propylthiouracil and methimazole (MMI)] are the most frequent causes of drug-induced ANCA-associated vasculitis [6–8]. Positive myeloperoxidase antineutrophil cytoplasmic antibody (MPO-ANCA) is reported in 4.1–64% of patients given antithyroid drugs, with many of them being asymptomatic [9–12]. Hypertrophic pachymeningitis (HP) is an inflammatory disease characterized by thickening of the dura mater, and can develop in association with ANCA-associated vasculitis [13–15]. ANCA-related HP is the most common form of HP, representing as many as 34.0% of all HP cases in Japan [16].

There have been few reported cases of optic neuropathy due to HP caused by ANCA-associated vasculitis, suspected to be drug related [17]. We present an optic neuropathy caused by HP with ANCA-associated vasculitis in a patient with Graves’ disease, where the patient had a good visual outcome after stopping antithyroid drug use and undergoing immunosuppression therapy.

## Case presentation

**Fig. 1:**
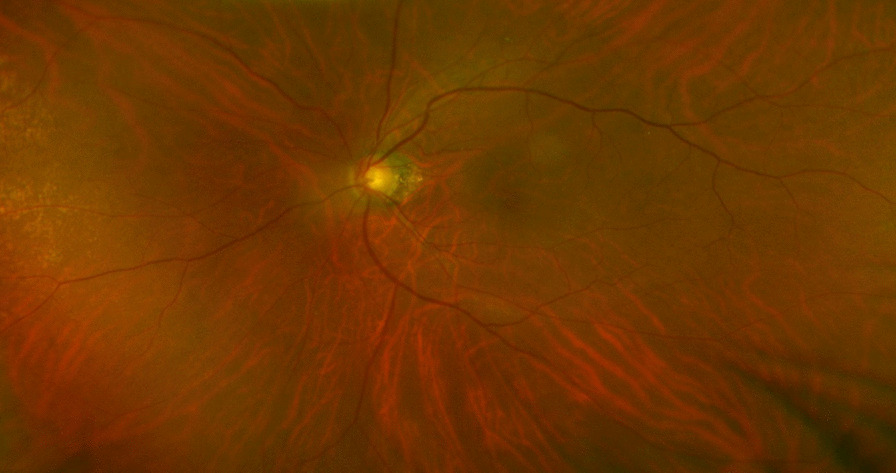
Fundus photograph of the left eye at the first visit. There were no findings that demonstrated that the best-corrected visual acuity of this eye was “light perception” in the fundus. No swelling was seen in the optic disc

**Fig. 2:**
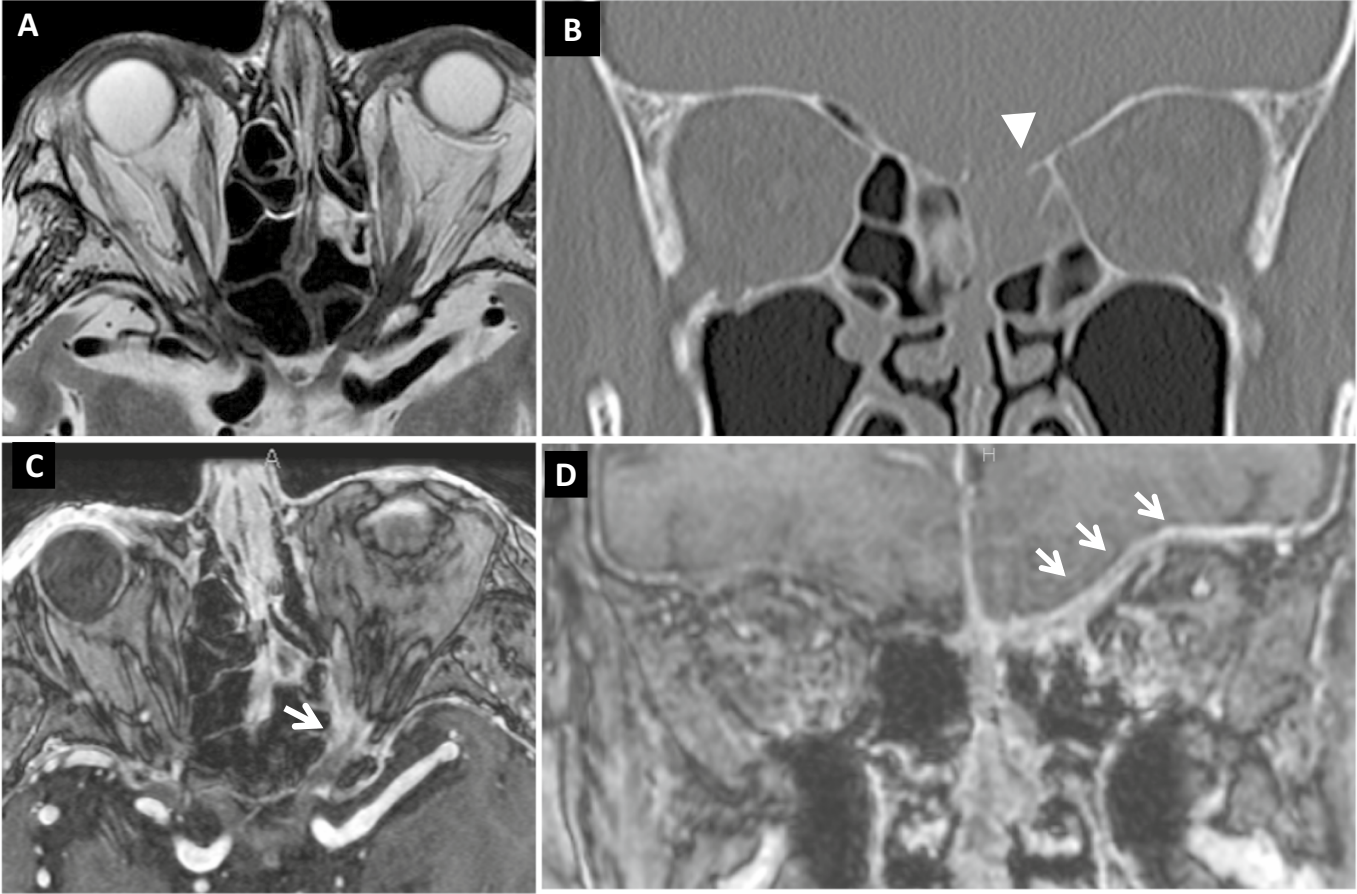
MRI and CT images. **A** shows a plain MRI image with mild enlargement of the external eye muscle in both eyes, with no findings of direct compression to the optic nerve by enlarged extraocular muscles nor stretching of the optic nerve by proptosis. **B** shows a coronal section of a plain head CT scan demonstrating the left ethmoid sinus filled with soft tissue and bone destructions in anterior cranial base (white arrow head). **C** shows contrast-enhanced MRI demonstrating the left optic nerve surrounded by enhancement (white arrow), suggesting inflammation at the optic canal. **D** shows a coronal section of contrast-enhanced MRI demonstrating thickened mucous membranes in the left ethmoid sinus and enhanced thicken dura of the bottom of left frontal cortex (white arrows)

**Fig. 3:**
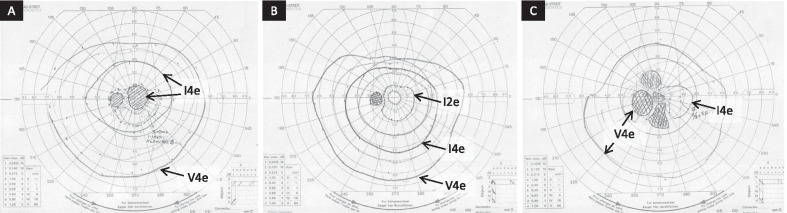
Results of Goldman visual field in the left eye with clinical course. A central scotoma is seen at 6 days after administering systemic steroids (**A**). The central scotoma disappeared in the 9 weeks after starting systemic steroids (**B**). Scotomas are seen when optic neuropathy relapse occurred at 6 months after starting treatment (**C**)

A 79-year-old Japanese man with acute vision loss in the left eye was referred to us in February 2015. He had a 24-year history of Graves’ disease and was taking MMI (starting dose of 30 mg/day in 1991, which was gradually reduced to 5 mg/alternate day by the time of his first visit) and levothyroxine (25 µg/day since 2013). He also had chronic obstructive pulmonary disease with pulmonary emphysema. The best-corrected visual acuity (BCVA) was 0.8 in the right eye and light perception in the left eye, and he had a relative afferent pupillary defect in the left eye. Extraocular muscle movements were normal, and exophthalmometer (Hertel) measurements were 20 mm bilaterally. There were no findings indicative of vision loss in the intermediate optic media or fundus of the left eye (Fig. 1). Although his thyroid-stimulating hormone level was low [0.11 µIU/mL (reference value: 0.50–5.00 µIU/mL)], his thyroid function was almost normal [free T3: 2.55 pg/mL (2.3–4.0 pg/mL), free T4: 1.73 ng/dL (0.9–1.7 ng/dL)]. Although plain magnetic resonance imaging (MRI) demonstrated mild enlargement of the extraocular muscles in both eyes, there was no compression or stretching of the optic nerve, which would have been suspicious of thyroid optic neuropathy (Fig. 2A). A computed tomography (CT) scan of the head showed the left ethmoid sinus to be filled with soft tissue and destruction of the upper ethmoid sinus wall, which included the anterior cranial base (Fig. 2B). A contrast-enhanced MRI revealed thickened mucous membranes in the left ethmoid sinus, enhanced dura from the left orbital apex to the anterior cranial base, and a mild enhancement of the optic nerve surrounded by enhancement at the orbital apex (Fig. 2C, D). We suspected optic neuropathy due to HP and infectious rhinogenic optic neuropathy due to infectious sinusitis. Inflammatory markers were high based on the laboratory data: white blood cells 8680/µL (3500–8500/µL) with 76.1% neutrophils (40–70%); C-reactive protein (CRP) 2.40 mg/dL (≤ 0.3 mg/dL); erythrocyte sedimentation rate 31 mm/hour (≤ 10 mm/hour) and 53 mm/2 hours (≤ 25 mm/2 hours); however, markers related to infection did not show a remarkable change [procalcitonin: 0.07 ng/mL (≤ 0.49 ng/mL), β-d-glucan: < 6.0 pg/mL (0–10.9 pg/mL)]. We performed further examinations for suspected HP, and MPO-ANCA was 19.5 U/mL (< 3.5 U/mL), proteinase 3-ANCA was <1.0 U/mL (< 3.5 U/mL), and IgG-4 was 42.1 mg/dL (4.8–105 mg/dL). A whole-body CT scan showed no malignancy, but pulmonary nodule lesions and a blood clot in the left lower leg were detected. Proteinuria and hematuria were both positive, and he had bloody nasal discharge. We consulted rheumatologists and pulmonologists, who indicated that the findings were consistent with ANCA-associated vasculitis. After ruling out diseases that could lead to HP, such as syphilis, aspergillosis, and tuberculosis. Serological tests and interferon gamma release assays established a diagnosis of optic neuropathy secondary to HP with ANCA-associated vasculitis. The clinical findings met the diagnostic criteria for GPA [18, 19] involving the upper respiratory tract, respiratory organs, and kidneys. We diagnosed optic neuropathy due to HP secondary to GPA. High-dose steroid pulse therapy (1000 mg of daily methylprednisolone for 3 days) was initiated, followed by 50 mg (1 mg/kg body weight) of daily oral prednisolone (PSL), which was subsequently tapered. MMI was discontinued, since ANCA-associated vasculitis could be induced by antithyroid drugs. Six days into treatment, the patient’s BCVA recovered to 0.03. Nine days into treatment, Goldmann perimetry (GP) showed a central scotoma in the left eye (Fig. 3A). Visual recovery was insufficient, so an endoscopic sinus biopsy was done on the same day to rule out malignancy and confirm the diagnosis. A biopsy of the posterior ethmoid sinus mucosa and nasal septal mucosa showed severe infiltration of chronic inflammatory cells, including neutrophils. There were no findings indicative of malignancy. While perivascular hyperplasia of connective tissue was observed, no apparent vasculitis or granuloma was seen. The BCVA of the left eye recovered to 0.5 within 3 weeks after the start of the treatment, and a contrast-enhanced MRI revealed rapid regression of enhanced dura from the left orbital apex to the anterior cranial base and enhancement of the optic nerve became unclear, however, enhancement surrounding the optic nerve remained (Additional file 1: Figs. A and B). The BCVA of the left eye recovered to 0.9 within 6 weeks after the start of treatment, and the central scotoma resolved 7 weeks following surgery (Fig. 3B). Thyroid function was stable despite discontinuation of MMI.

Six months after starting the treatment, when PSL was tapered to 15 mg, the patients BCVA decreased and GP revealed scotomas in the left eye (Fig. 3C). A contrast-enhanced MRI, taken for follow-up 1 week before the patient realized his vision had deteriorated, showed thickened enhanced dura from the left orbital apex to the anterior cranial base, and the optic nerve surrounded by enhancement at the orbital apex (Additional file 1: Figs. C and D). Furthermore, mild enhancement of the optic nerve appeared to recur (Additional file 1: Figs. A and B). There was no remarkable change in laboratory findings, including MPO-ANCA and CRP levels. We started a second round of high-dose steroid pulse therapy (1000 mg of daily methylprednisolone for 3 days), followed by a PSL taper. We added 4 mg/week of methotrexate (MTX) to the PSL taper and discontinued it after 3.5 years. He had no relapse after the addition of MTX, and MPO-ANCA levels were within normal limits until 4 years after onset. Three months after his 4-year follow-up, he died from hypoxic ischemic encephalopathy due to pulmonary emphysema.

## Discussion and conclusions

We herein report a case of optic neuropathy secondary to HP with ANCA-associated vasculitis, which was suspected to be related to MMI. The patient had good visual recovery after immunosuppression therapy and MMI discontinuation. Low-dose MTX was effective to keep the patient in remission.

Although we first suspected thyroid optic neuropathy, suspicious findings of thyroid optic neuropathy were not seen, and the presence of thickened dura from the left orbital apex to the anterior cranial base with the coronal section of a contrast-enhanced MRI pointed to optic neuropathy due to HP. Though cerebrospinal fluid examination could have ruled out infection, the risks outweighed the benefits of the procedure. Since HP and sinusitis could be associated with ANCA-associated vasculitis, [15] and other causes of HP were ruled out, we diagnosed the current case as optic neuropathy due to ANCA-related HP. More than 80% of MPO-ANCA-positive HP patients were reported to have GPA, an ANCA-associated vasculitis subtype [20]. An orbital tissue biopsy when the patient first presented, could have confirmed the diagnosis histologically. However, we started steroid pulse therapy immediately, on a priority basis, after diagnosing optic neuropathy due to noninfectious HP, and could not confirm our diagnosis histologically. Nevertheless, it was possible to diagnose the present case as GPA according to the stepwise algorithm of Watt *et al.*, which was composed using the American College of Rheumatology (ACR) criteria for vasculitis [21–23] and the Chapel Hill Consensus Conference definition of vasculitis [24] to classify ANCA-associated vasculitis and polyarteritis nodosa [19]. Even though the histological finding could not be shown, three of the four criteria (nasal inflammation with bloody nasal discharge, abnormal chest radiograph showing nodules, and hematuria) were present in the current case, indicating GPA. Furthermore, the current case also matched the GPA diagnostic criteria from the study group of systemic vascular disorders in Japan; GPA could be diagnosed using the clinical findings of more than three major organs (upper respiratory tract, lungs, kidneys, and vessels) [18].

ANCA-associated vasculitis can be induced by antithyroid drugs, even after several years of use and at low doses. MPO-ANCA is a marker of antithyroid drug-induced ANCA-associated vasculitis [17, 25]. In this case, MMI was taken for 24 years at an initial daily dose of 30 mg; this was gradually reduced to 5 mg/alternate day by the time he presented for examination at our clinic. Noh *et al.* reported that ANCA-associated vasculitis secondary to antithyroid drugs involved single or multiple organs, such as the kidneys, respiratory organs, skin, joints, eyes, muscles, brain, and nerves [17]. In the current case, the respiratory organs, brain, kidneys, and nerves were involved. Although it is difficult to identify whether the ANCA-associated vasculitis is drug-induced, the possibility must be kept in mind to make a decision about the discontinuation of the drug usage.

We suspected that ANCA-associated vasculitis was antithyroid drug related, and that prognosis could be improved by discontinuation of the drug. Thus, we administered only steroids for immunosuppression and planned to taper the PSL. The following mechanisms of optic nerve damage have been hypothesized: vasculitic infarction [26, 27], nonvasculitic optic nerve inflammation, or spread of inflammation from the adjacent sinuses [28, 29]. Moreover, some GPA-related isolated neuropathy cases were suspected to be caused by granulomatous optic neuropathy located within the optic nerve sheath [5]. As MRI showed mild enhancement of the optic nerve and good visual recovery after treatment in the current case, we considered spread of inflammation and compression due to thickened dura by HP were the main cause.

Upon recurrence of optic neuropathy, we added low-dose MTX to the PSL taper, which proved to be effective against future relapse. Cyclophosphamide (CYP) has also been used as induction therapy for ANCA-associated vasculitis, but a previous clinical trial demonstrated that the efficacy of MTX and CYP were equivalent with regard to the remission rates in patients with early ANCA-associated vasculitis [30]. As MTX has lower toxicity than CYP in elderly patients [20], we selected MTX in this treatment regimen.

While an ANCA titer was previously reported to be a predictable factor for ANCA-associated vasculitis relapse with renal involvement, but not without renal involvement [31], another MPO-ANCA-positive HP case report found that disease activity was related to the MPO-ANCA titer during long-term follow-up [32]. Here, the MPO-ANCA titer was within normal limits at the time of recurrence. An ANCA titer can sometimes predict relapse, but it is not always related to disease activity. Thus, laboratory results and symptoms of ANCA-associated vasculitis-involved organs must be carefully monitored to keep the vasculitis in remission.

In conclusion, HP secondary to ANCA-associated vasculitis, suspected to be antithyroid drug related, needs to be considered in patients with Graves’ disease when optic neuropathy is present but optic nerve compression by extraocular muscles is not obvious. A good visual prognosis can be achieved by discontinuing antithyroid drugs and initiating immunosuppressive therapy.

## Supplementary Information

**Additional file 1. Figure**. Contrast-enhanced MRI images during remission and recurrence. A, B: MRI images at the time of remission, taken 2 weeks after the start of treatment. C, D: MRI images taken for follow-up, one week before the patient realized his vision had deteriorated for the recurrence. White arrows indicate enhanced dura of the bottom of left frontal cortex (B, D) . The enhancement in (D) appears thicker and stronger than that of in (B). White arrow head points enhancement surrounding mildly enhanced the optic nerve (C).

## Data Availability

The data used in the case report are available on reasonable request.

## Abbreviations

ACR: The American College of Rheumatology
ANCA: Antineutrophil cytoplasmic antibody
BCVA: Best-corrected visual acuity
CRP: C-reactive protein
CT: Computed tomography
GP: Goldmann perimetry
GPA: Granulomatosis with polyangiitis
HP: Hypertrophic pachymeningitis
MMI: Methimazole
MPO: Myeloperoxidase
MRI: Magnetic resonance imaging
MTX: Methotrexate
PSL: Prednisolone
