# Supplementary material for: Optic neuropathy secondary to granulomatosis with polyangiitis in a patient with Graves’ disease: a case report
Source: J Med Case Rep. 2021 Dec 29;15:618. doi: 10.1186/s13256-021-03207-4 (PMC8715570; doi:10.1186/s13256-021-03207-4)
Supplement: Supplementary file 1 — Additional file 1. Figure. Contrast-enhanced MRI images during remission and recurrence. A, B: MRI images at the time of remission, taken 2 weeks after the start of treatment. C, D: MRI images taken for follow-up, one week before the patient realized his vision had deteriorated for the recurrence. White arrows indicate enhanced dura of the bottom of left frontal cortex (B, D) . The enhancement in (D) appears thicker and stronger than that of in (B). White arrow head points enhancement surrounding mildly enhanced the optic nerve (C). [file 13256_2021_3207_MOESM1_ESM.pdf]

Supplemental Figure

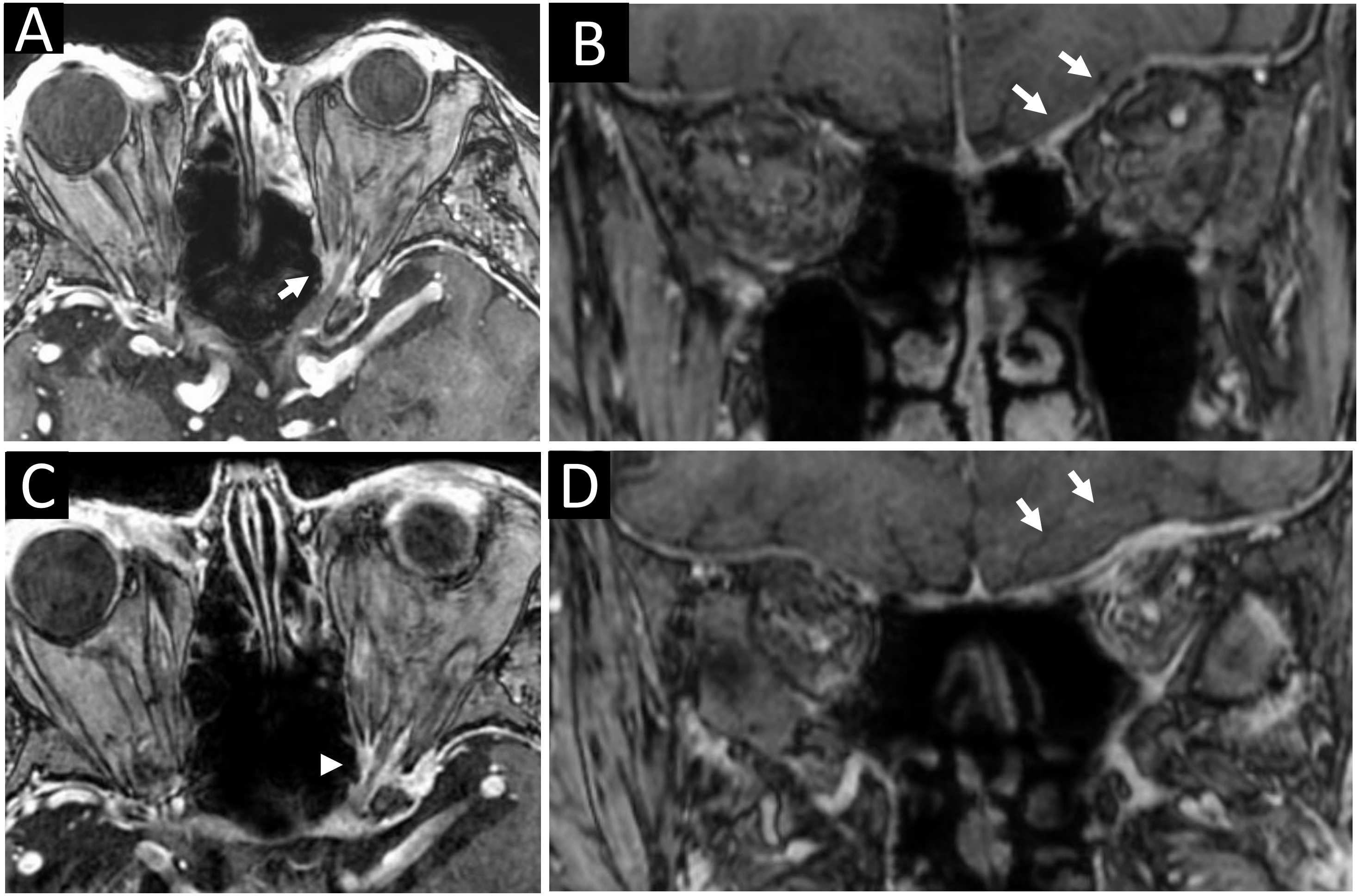

Additional file 1: Figure. Contrast-enhanced MRI images during remission and recurrence
A, B: MRI images at the time of remission, taken 2 weeks after the start of treatment.
C, D: MRI images taken for follow-up, one week before the patient realized his vision had deteriorated for the recurrence.
White arrows indicate enhanced dura of the bottom of left frontal cortex (B, D) . The enhancement in (D) appears thicker and stronger than that of in (B).
White arrow head points enhancement surrounding mildly enhanced the optic nerve (C).
